# Early Post-Transplant Urinary EGF as a Potential Predictor of Long-Term Allograft Loss in Kidney Transplant Recipients

**DOI:** 10.3389/ti.2025.15061

**Published:** 2025-10-20

**Authors:** Antoine Créon, Lise Morin, Virginia Garcia, Laila Aouni, Marion Rabant, Fabiola Terzi, Dany Anglicheau

**Affiliations:** 1 Department of Nephrology and Kidney Transplantation, Necker Hospital, AP-HP, Paris, France; 2 Department of Medical Epidemiology and Biostatistics, Karolinska Institutet, Stockholm, Sweden; 3 Université Paris Cité, INSERM U1151, CNRS UMR8253, Institut Necker Enfants Malades (INEM), Paris, France; 4 Department of Pathology, Necker Hospital, AP-HP, Paris, France

**Keywords:** fibrosis, kidney transplant failure, allograft dysfunction, survival analysis, epidermal growth factor receptor

## Abstract

Improved biomarkers are needed to enhance prognostication in kidney transplantation. We evaluated urinary Epidermal Growth Factor (uEGF) as a predictor of long-term allograft loss. We conducted a prospective, single-center cohort study of 290 adult kidney transplant recipients with uEGF measured 3 months post-transplant. The primary outcome was allograft loss, defined as return to dialysis or pre-emptive re-transplantation. Multivariable cause-specific Cox models assessed the independent association between uEGF and allograft loss. Model performance was compared to an existing prediction model using 7-year time-dependent AUC and Akaike Information Criterion (AIC), with internal validation via bootstrap resampling. Temporal validation was performed in an independent cohort of 203 patients. uEGF correlated with markers of chronic injury, including eGFR, donor age, and interstitial fibrosis. After a median 8.8- year follow-up, lower uEGF was independently associated with allograft loss (adjusted HR 0.19; 95% CI, 0.11−0.32). Adding uEGF to the existing prediction model improved discrimination (AUC 0.72 vs. 0.63) and reduced AIC (383 vs. 394). While results were robust to internal validation, temporal validation did not show an independent association of uEGF with allograft loss. These findings suggest uEGF may provide independent prognostic value, but further studies in larger and more diverse cohorts are needed to confirm its clinical utility.

## Graphical Abstract

**Figure:**
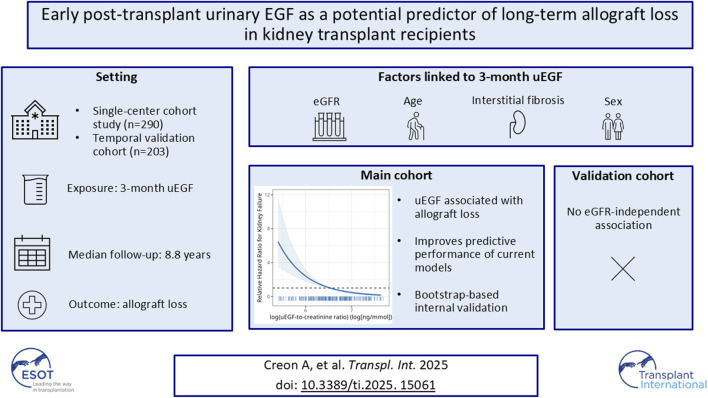

## Introduction

Kidney transplantation is the standard-of-care for kidney failure. However, dialysis is still the predominant therapy in many countries [1]. One limiting factor to access kidney transplantation is an insufficient number of kidney donors to meet the needs [2]. As such, improving allografts long-term outcomes is paramount. Although marked advances in acute rejection prevention and treatment have been made over the past decades, they failed to translate into meaningful improvement in kidney allograft survival [3]. International societies acknowledged the need for better predictors of long-term outcomes, to guide patients’ monitoring and tailor therapeutic strategies, but also to be implemented in clinical trials as surrogate endpoints for efficacy [4]. As causes of late allograft loss are heterogenous, efforts have been made to include known predictors of poor outcome into prognostic scores, yielding much better accuracy than when taken individually [5]. A complementary approach involves developing innovative markers able that better capture the risk of long-term kidney injury, irrespective of its cause. The overall burden of chronic kidney damage has been strongly associated with allograft loss, regardless of the underlying etiology [6]. Therefore, detecting subclinical molecular mechanisms involved in fibrotic processes may offer a valuable strategy to improve the prediction of long-term allograft failure.

Several animal models have highlighted the key role of the Epidermal Growth Factor Receptor (EGF-R) pathway in mediating kidney fibrosis [7]. While its activation has been linked to CKD progression, it is paradoxically the reduction of its ligand, urinary EGF (uEGF), that has been associated with disease progression. uEGF has been shown to be decreased in renal biopsies of patients with chronic kidney disease (CKD) and was associated with early decline in glomerular filtration rate (eGFR) in patients with CKD, transplant recipients, and in the general population [8–11]. This observation suggests that uEGF may have a protective role in kidney physiology, or that its levels may serve as a surrogate marker of preserved nephron mass.

We hypothesized that uEGF may reflect a subclinical signaling process involved in accelerated allograft damage, that would eventually translate into reduced long-term allograft survival. In the current study, we prospectively evaluated the association between early post-transplant uEGF and long-term allograft survival in a cohort of kidney transplant recipients. We aimed to assess its association with known prognostic factors, and whether uEGF improves long-term allograft loss prediction. Finally, we evaluated the incremental value of uEGF through internal validation using bootstrap resampling, and examined whether its association with allograft loss could be replicated in a temporally distinct validation cohort from the same center.

## Materials and Methods

The study followed the Transparent Reporting of a multivariable prediction model for Individual Prognosis Or Diagnosis (TRIPOD) guidelines for the development of prediction models [12].

### Population

All consecutive adults who received a kidney transplant in our center from June 13th, 2009, to June, 6^th^, 2012 were considered for inclusion in this prospective, longitudinal, single-center cohort study. The retrospective validation cohort was a random sample of patients transplanted from June 10^th^ 2012 to November 4^th^ 2020, for whom stored urine samples were available. Selection was performed by a laboratory technician blinded to clinical characteristics. Patients with active HIV or Hepatitis C virus infection, allograft loss before 3 months post-transplant, death or lost to follow-up before 6 months post-transplant were excluded. The present study was approved by the Ethics Committee of Ile-de-France XI (#13016). All participants provided written informed consent.

### Kidney Biopsies

Protocol biopsies were performed at months 3 and 12 post-transplant. Indication biopsies were performed for clinical indications. In addition to light microscopy evaluation, C4d immunohistochemical staining was systematically performed (rabbit anti-human monoclonal anti-C4d, Clinisciences, Nanterre, France, 1:200 dilution). Kidney allograft biopsies were classified using the Banff 2017 classification [13].

### Immunosuppression

All but 11 patients received induction therapy with rabbit anti-thymocyte globulin (Thymoglobuline, Sanofi, Marcy l’Etoile, France [n = 152]) or basiliximab (Simulect, Novartis Pharma AG, Basel, Switzerland [n = 127]). Maintenance immunosuppression consisted of a three-drug regimen that included steroids (n = 290) and mycophenolate mofetil (n = 290) with a calcineurin inhibitor (n = 287, ciclosporine n = 59 and tacrolimus n = 228) or everolimus (n = 3). All patients with Donor Specific anti-HLA Antibodies (DSA) at time of transplantation (n = 128) received four courses of intravenous immunoglobulines in addition to the three-drug regimen. DSA-positive patients with mean fluorescence intensity (MFI) > 1,000 at day 0 (n = 55) received additional prophylactic rituximab therapy (Mabthera, Roche Pharmaceuticals, Basel, Switzerland), together with plasmapheresis.

### Samples Collection and Analysis

Urine samples were collected at month 3 post-transplant, both in the derivation and validation cohort. Samples were centrifugated at 1,000 g for 10 min, within 4 h of collection. The supernatant was collected after centrifugation and stored with protease inhibitors (cOmplete™, EDTA-free Protease Inhibitor Cocktail, Roche, Basel, Switzerland) at −80 °C. uEGF was quantified by ELISA (human EGF Quantikine EG00 kit, R&S Systems, Minneapolis, USA) and standardized to urine creatinine.

### Covariates and Outcomes

Covariates were prospectively collected from the medical records by research assistants. Baseline covariates were measured at 3 months post-transplant. Glomerular filtration rate was estimated using the 2009 CKD-EPI equation [14]. The outcomes were allograft loss, defined as definitive return to dialysis or pre-emptive kidney retransplantation, and death. Patients were followed from inclusion (at month 3 post-transplantation) to the date of allograft loss, death or administrative censoring (March 11th of 2024), whichever occurred first.

### Statistical Analyses

Continuous variables were described using mean and standard error or median and interquartiles intervals if not normally distributed. Median follow-up was calculated using the inverse Kaplan-Meier method. For descriptive purposes, uEGF was also categorized into tertiles.

A random forest regression was performed to identify the baseline variables predicting uEGF at 3 months post-transplant. The model’s parameters were optimized by 10-fold cross-validation. A grid of 100 parameter combinations was tested, with a number of predictors to be randomly sampled at each split (mtry) between 10 and 34, a number of trees in the ensemble between 500 and 1,500, and a minimum number of data points in a node that are required for the node to be split further (min_n) between 1 and 34. The optimal combination of parameters was selected based on root mean square error (RMSE), and the model was updated with these optimized parameters (mtry = 19, trees = 1,198, min_n = 20). Linear regression analysis was also performed to evaluate the association between uEGF at month 3 post-transplant and the patients’ characteristics. For continuous variables, the slope corresponds to the variation in uEGF for one unit variation of the independent variable. For categorical variables, it corresponds to the difference in uEGF means between the category of interest and the reference one. Finally, we report the Spearman’s rank correlation coefficient between uEGF and eGFR to avoid assumptions of normality and linearity in their relationship.

Cumulative incidence functions were estimated using the Aalen-Johansen estimator, accounting for the competing risk of death. Univariable cause-specific Cox regression analyses were performed to assess the association between allograft loss and all studied variables. For categorical variables, proportional hazards assumption was checked with a Schoenfeld residuals test. Log-linearity was assessed using the cubic splines method. Before integration in survival analyses, uEGF-to-creatinine and protein-to-creatinine ratios were log-transformed. We used cause-specific Cox models rather than Fine and Gray subdistribution hazard models to ensure consistency with the iBox scoring system, which is based on a Cox model.

To assess the robustness of the association between uEGF and allograft loss, several adjustment strategies were used. First, a stepwise forward selection procedure was applied: starting from a null model, covariates were sequentially added based on statistical significance, with the most strongly associated covariate added at each step. The selection stopped when a maximum of one covariate per 10 events was reached or when no additional covariate met the significance threshold (*p* < 0.05). Second, a model was built by selecting covariates most associated with uEGF using random forest variable importance rankings. Finally, a model was constructed by adding uEGF to the allograft loss risk score (ALRS) described by Loupy et al., which is the reference model for allograft loss prediction [5].

The models’ discrimination ability was evaluated using the time-dependent area under the curve (AUC) at 7 years, as risks of allograft loss beyond 7 years could not be derived from the original ALRS publication (see Supplementary Methods). Discrimination was assessed for both the ALRS model and the extended model including uEGF, and their 7-year AUC was compared as in Blanche et al. [15]. Confidence intervals for the AUC were obtained using the estimated standard error of the AUC and assuming approximate normality. The Akaike Information Criterion (AIC) was also used to compare model fit, with lower AIC values indicating a better balance between complexity and goodness of fit. Harrell’s C-index was not used, as it may be less appropriate in this setting where risk predictions are made at a specific time point [16]. To account for overfitting, internal validation was performed using 1,000 bootstrap resamples. Discrimination and calibration were optimism- corrected, with the latter assessed visually using a calibration plot comparing predicted and observed 7-year risks across quantiles of predicted risk. To reflect the original ALRS publication, observed 7-year risks were estimated using the Kaplan -Meier method rather than the Aalen -Johansen estimator when assessing calibration. Only complete cases were used in the analysis. Data management, statistical analyses and graphics were performed using R software 4.1.2.

## Results

### Characteristics of the Cohort

**FIGURE 1:**
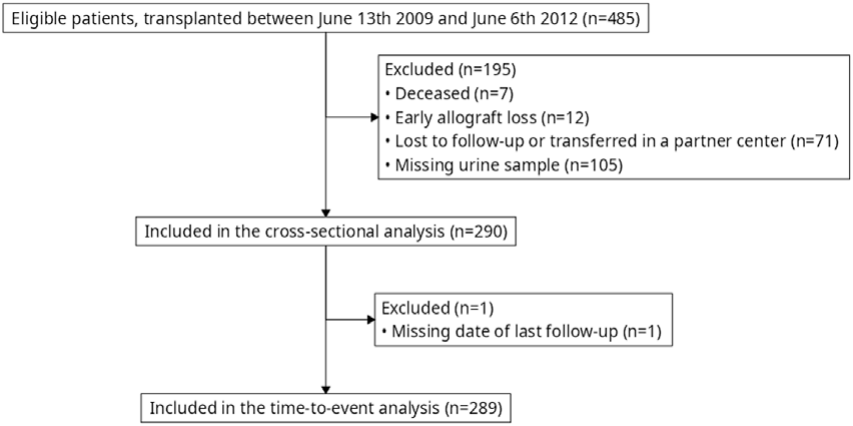
Flowchart.

Between June 13^th^ 2009 and June 6^th^ 2012, 485 patients were transplanted in our center (Necker Hospital, Paris, France). Among them, 290 were included in the present study. A 3-month kidney biopsy that yielded adequate results was available for 274 patients. Donor specific anti-HLA antibodies (DSA) at months 3 were available for 277 patients (Figure 1).

The characteristics of the cohort are detailed in Table 1. Kidney transplant recipients had mean age of 50.8 years at the time of surgery, and 59% of them were males. The main causes of kidney failure were glomerulonephritis (26.6%), unknown nephropathy (20.7%), autosomal dominant polycystic kidney disease (14.8%) and diabetes (10.7%). Among donors, 24.5% were living donors, 39% expanded-criteria deceased donors (ECD) and 36.6% standard-criteria deceased donors. 44.1% had pre-transplant DSA and 14.1% had a previous kidney transplant. Induction therapy consisted of Basiliximab (43.8%) or anti-thymocyte globulin (52.4%). Maintenance therapy was always a triple therapy, including steroids and mycophenolate mofetil, associated with tacrolimus (78.6%), ciclosporine (20.3%) or everolimus (1%). At 3 months, the mean (SD) estimated glomerular filtration rate (eGFR) was 56.3 (23) mL/min/1.73 m^2^ and the median (Q1-Q3) proteinuria was 0.02 (0.01–0.04) g/mmol. The uEGF:Cr ratio was normally distributed when log-transformed (Supplementary Figure S1).

**TABLE 1:** characteristics at baseline.

Baseline Characteristics	Overall, N = 290^a^	uEGF tertiles			Missing
		113.34–546.49 ng/mmol, N = 97^a^	546.49–916.26 ng/mmol, N = 96^a^	916.26–3,790.27 ng/mmol, N = 97^a^	
Donor age	54 (18)	61 (14)	55 (17)	47 (21)	0 (0%)
Donor type					0 (0%)
Standard-Criteria	106 (37%)	28 (29%)	33 (34%)	45 (46%)	
Expanded-Criteria	113 (39%)	59 (61%)	31 (32%)	23 (24%)	
Living Donor	71 (24%)	10 (10%)	32 (33%)	29 (30%)	
Recipient age	51 (15)	52 (15)	50 (15)	51 (15)	0 (0%)
Recipient sex					0 (0%)
Male	171 (59%)	66 (68%)	59 (61%)	46 (47%)	
Female	119 (41%)	31 (32%)	37 (39%)	51 (53%)	
Recipient ethnicity					0 (0%)
Caucasian	183 (63%)	58 (60%)	60 (63%)	65 (67%)	
Black	52 (18%)	20 (21%)	19 (20%)	13 (13%)	
North-african	46 (16%)	18 (19%)	14 (15%)	14 (14%)	
Other	9 (3.1%)	1 (1.0%)	3 (3.1%)	5 (5.2%)	
Cause of kidney failure					0 (0%)
Diabetes	31 (11%)	10 (10%)	12 (13%)	9 (9.3%)	
Glomerulonephritis	77 (27%)	25 (26%)	28 (29%)	24 (25%)	
Hypertensive	19 (6.6%)	6 (6.2%)	5 (5.2%)	8 (8.2%)	
Tubulo-interstitial	24 (8.3%)	8 (8.2%)	9 (9.4%)	7 (7.2%)	
Autosomal dominant polycystic kidney disease	43 (15%)	14 (14%)	12 (13%)	17 (18%)	
Unknown	60 (21%)	19 (20%)	19 (20%)	22 (23%)	
Other	36 (12%)	15 (15%)	11 (11%)	10 (10%)	
Recipient body mass index	24.4 (4.7)	24.8 (5.0)	24.2 (4.1)	24.4 (4.9)	0 (0%)
Prior kidney transplant	41 (14%)	20 (21%)	7 (7.3%)	14 (14%)	0 (0%)
Cold ischaemia time (min)	960 (547–1,423)	1,100 (797–1,602)	887 (158–1,380)	900 (176–1,346)	3 (1.0%)
HLA A/B/DR mismatch	5.00 (4.00–5.00)	5.00 (4.00–6.00)	5.00 (4.00–5.00)	4.00 (4.00–5.00)	0 (0%)
ABO compatibility	260 (90%)	90 (93%)	86 (90%)	84 (87%)	0 (0%)
Pre-existing anti-HLA donor-specific antibody					0 (0%)
MFI <500	162 (56%)	56 (58%)	51 (53%)	55 (57%)	
MFI 500–1,000	73 (25%)	23 (24%)	23 (24%)	27 (28%)	
MFI 1000–3,000	38 (13%)	13 (13%)	16 (17%)	9 (9.3%)	
MFI >3,000	17 (5.9%)	5 (5.2%)	6 (6.3%)	6 (6.2%)	
DSA Immunodominant MFI at M3					13 (4.5%)
<500	124 (45%)	40 (43%)	40 (43%)	44 (48%)	
500–3,000	126 (45%)	42 (45%)	43 (47%)	41 (45%)	
3,000–6,000	12 (4.3%)	4 (4.3%)	5 (5.4%)	3 (3.3%)	
>6,000	15 (5.4%)	7 (7.5%)	4 (4.3%)	4 (4.3%)	
Induction immunosuppression					0 (0%)
Anti-thymocyte globulin	152 (52%)	56 (58%)	42 (44%)	54 (56%)	
Basiliximab	127 (44%)	38 (39%)	48 (50%)	41 (42%)	
No induction	11 (3.8%)	3 (3.1%)	6 (6.3%)	2 (2.1%)	
Maintenance immunosuppression					0 (0%)
Steroids, Mycophenolate Mofetil and Ciclosporine	59 (20%)	23 (24%)	18 (19%)	18 (19%)	
Steroids, Mycophenolate Mofetil and Tacrolimus	228 (79%)	72 (74%)	77 (80%)	79 (81%)	
Steroids, Mycophenolate Mofetil and Everolimus	3 (1.0%)	2 (2.1%)	1 (1.0%)	0 (0%)	
Delayed graft function	61 (21%)	32 (33%)	21 (22%)	8 (8.2%)	0 (0%)
Estimated glomerular filtration rate at 3 months (ml/min/1.73 m^2^)	56 (23)	40 (14)	57 (18)	72 (22)	0 (0%)
Proteinuria at 3 months (g/mmol)	0.02 (0.01–0.04)	0.02 (0.02–0.04)	0.02 (0.01–0.03)	0.03 (0.02–0.04)	1 (0.3%)
Glomerulitis (g)					16 (5.5%)
0	215 (78%)	69 (77%)	68 (76%)	78 (83%)	
1	44 (16%)	13 (14%)	19 (21%)	12 (13%)	
2	11 (4.0%)	6 (6.7%)	1 (1.1%)	4 (4.3%)	
3	4 (1.5%)	2 (2.2%)	2 (2.2%)	0 (0%)	
Interstitial inflammation (i)					16 (5.5%)
0	261 (95%)	87 (97%)	85 (94%)	89 (95%)	
1	10 (3.6%)	2 (2.2%)	4 (4.4%)	4 (4.3%)	
2	3 (1.1%)	1 (1.1%)	1 (1.1%)	1 (1.1%)	
3	0 (0%)	0 (0%)	0 (0%)	0 (0%)	
Total interstitial inflammation (ti)					16 (5.5%)
0	252 (92%)	76 (84%)	86 (96%)	90 (96%)	
1	14 (5.1%)	8 (8.9%)	4 (4.4%)	2 (2.1%)	
2	6 (2.2%)	4 (4.4%)	0 (0%)	2 (2.1%)	
3	2 (0.7%)	2 (2.2%)	0 (0%)	0 (0%)	
Tubulitis (t)					16 (5.5%)
0	237 (86%)	77 (86%)	80 (89%)	80 (85%)	
1	14 (5.1%)	6 (6.7%)	3 (3.3%)	5 (5.3%)	
2	7 (2.6%)	2 (2.2%)	2 (2.2%)	3 (3.2%)	
3	16 (5.8%)	5 (5.6%)	5 (5.6%)	6 (6.4%)	
Intimal arteritis (v)					16 (5.5%)
0	271 (99%)	90 (100%)	89 (99%)	92 (98%)	
1	2 (0.7%)	0 (0%)	1 (1.1%)	1 (1.1%)	
2	0 (0%)	0 (0%)	0 (0%)	0 (0%)	
3	1 (0.4%)	0 (0%)	0 (0%)	1 (1.1%)	
Peritubular capillaritis (ptc)					16 (5.5%)
0	235 (86%)	75 (83%)	81 (90%)	79 (84%)	
1	24 (8.8%)	7 (7.8%)	7 (7.8%)	10 (11%)	
2	12 (4.4%)	6 (6.7%)	1 (1.1%)	5 (5.3%)	
3	3 (1.1%)	2 (2.2%)	1 (1.1%)	0 (0%)	
Interstitial fibrosis (ci)					16 (5.5%)
0	156 (57%)	32 (36%)	51 (57%)	73 (78%)	
1	59 (22%)	22 (24%)	27 (30%)	10 (11%)	
2	32 (12%)	20 (22%)	7 (7.8%)	5 (5.3%)	
3	27 (9.9%)	16 (18%)	5 (5.6%)	6 (6.4%)	
Tubular atrophy (ct)					16 (5.5%)
0	154 (56%)	32 (36%)	51 (57%)	71 (76%)	
1	63 (23%)	24 (27%)	26 (29%)	13 (14%)	
2	34 (12%)	21 (23%)	9 (10%)	4 (4.3%)	
3	23 (8.4%)	13 (14%)	4 (4.4%)	6 (6.4%)	
C4d graft deposition (c4d)					16 (5.5%)
0	226 (82%)	72 (80%)	67 (74%)	87 (93%)	
1	27 (9.9%)	11 (12%)	12 (13%)	4 (4.3%)	
2	15 (5.5%)	5 (5.6%)	7 (7.8%)	3 (3.2%)	
3	6 (2.2%)	2 (2.2%)	4 (4.4%)	0 (0%)	
Vascular Fibrous Intimal Thickening (cv)					16 (5.5%)
0	101 (37%)	23 (26%)	33 (37%)	45 (48%)	
1	62 (23%)	17 (19%)	24 (27%)	21 (22%)	
2	82 (30%)	37 (41%)	25 (28%)	20 (21%)	
3	29 (11%)	13 (14%)	8 (8.9%)	8 (8.5%)	
Glomerular basement membrane double contours (cg)					16 (5.5%)
0	269 (98%)	89 (99%)	88 (98%)	92 (98%)	
1	5 (1.8%)	1 (1.1%)	2 (2.2%)	2 (2.1%)	
2	0 (0%)	0 (0%)	0 (0%)	0 (0%)	
3	0 (0%)	0 (0%)	0 (0%)	0 (0%)	
Arteriolar hyalinosis (ah)					16 (5.5%)
0	108 (39%)	21 (23%)	39 (43%)	48 (51%)	
1	116 (42%)	40 (44%)	38 (42%)	38 (40%)	
2	35 (13%)	20 (22%)	12 (13%)	3 (3.2%)	
3	15 (5.5%)	9 (10%)	1 (1.1%)	5 (5.3%)	

^a^ Mean (SD); n (%); Median (25%–75%).

### UEGF Is Associated With Long-Term Allograft Loss in Univariable Survival Analysis

**FIGURE 2:**
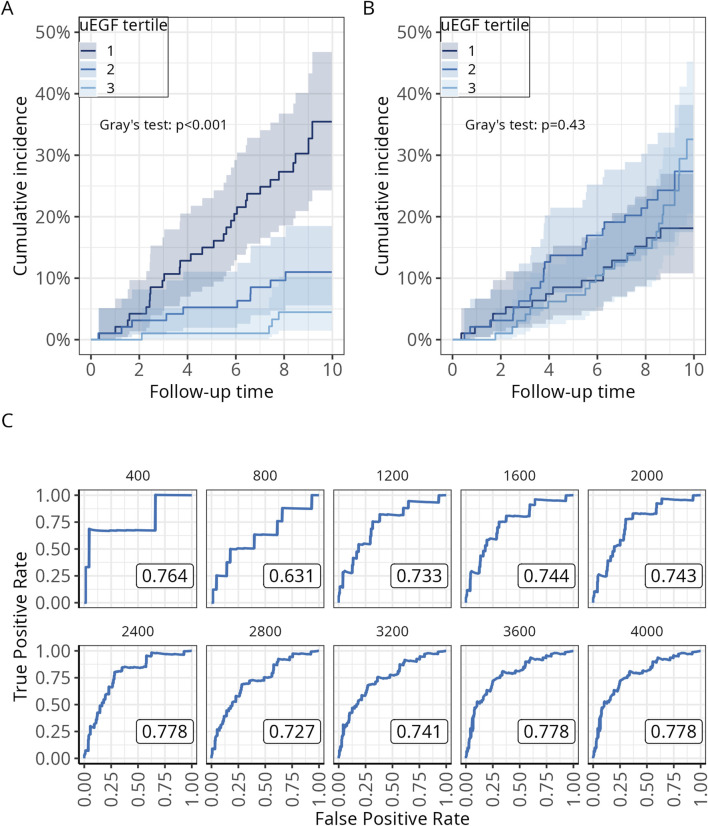
Relationship between allograft loss, death, and uEGF at 3 months post-transplant in univariable survival analysis. **(A)** Cumulative incidence curves for allograft loss across tertiles of uEGF at 3 months; **(B)** Cumulative incidence curves for all-cause death across tertiles of uEGF at 3 months; **(C)** Time-dependent ROC curves for uEGF at 3 months in diagnosing allograft loss, evaluated every 400 days following transplantation. The statistical significance of differences in survival across uEGF tertiles is assessed using Gray’s test. uEGF, urinary Epidermal Growth Factor. ROC, receiver operating curve.

After a median follow-up of 8.8 years, 43 patients experienced allograft loss and 62 died. Among those with allograft loss, 29 were in the first uEGF tertile, 10 in the second, and 4 in the third, with a significant difference in allograft failure cumulative incidence (Gray test, p-value <0.001) (Figure 2A). Patient survival across uEGF tertiles was comparable (Figure 2B). A time-dependent Receiver Operating Characteristic (ROC) curve analysis was performed to evaluate the discriminatory ability of uEGF levels measured at 3 months in distinguishing between patients who experienced allograft loss and those who did not over time. The model demonstrated good discrimination from 1200 to 4000 days post-transplant, with an area under the curve (AUC) ranging from 0.727 to 0.778 (Figure 2C).

### UEGF Is Associated With Markers of Allograft Chronic Damage

**FIGURE 3:**
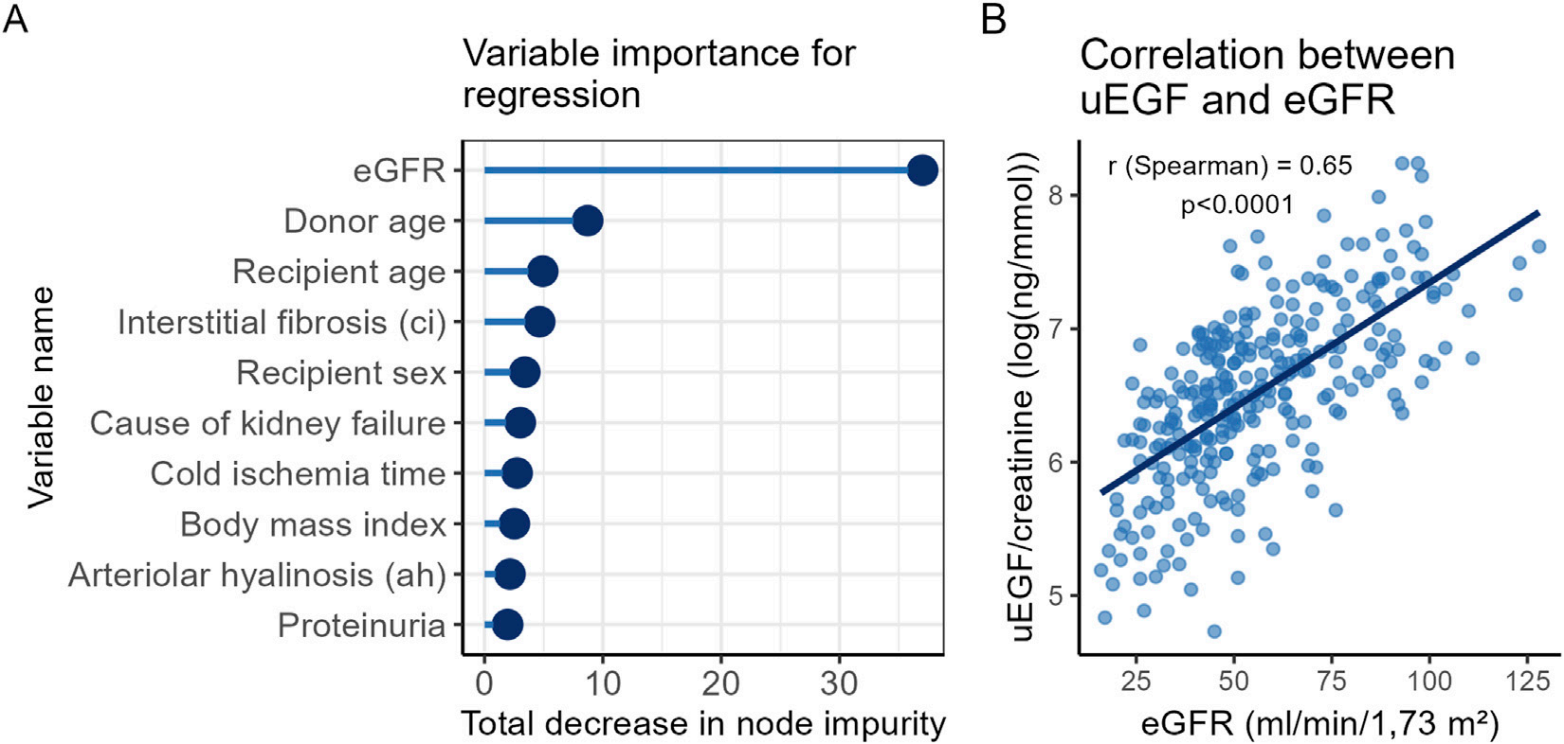
Covariates associated with uEGF levels at 3 months post-transplant. **(A)** Variable importance in explaining uEGF levels at 3 months post-transplant, measured by the total reduction in residual sum of squares in random forest regression analysis. Hyperparameters optimized by 10-fold cross validation were trees = 1,198, mtry = 19 and min_n = 20. Only the top 10 variables contributing most significantly to the model’s predictive performance are displayed. **(B)** Scatterplot of uEGF and eGFR distributions, with Sperman’s correlation coefficient. uEGF: urinary Epidermal Growth Factor. eGFR: estimated Glomerular Filtration Rate.

To identify the patient characteristics associated with uEGF concentrations, a random forest analysis was performed. The strongest association was observed with eGFR (Figure 2A), which also showed a high correlation with uEGF (correlation coefficient = 0.65, *p* < 0.0001; Figure 2B). To a lesser extent, uEGF was associated with recipient sex and markers of chronic allograft damage such as interstitial fibrosis, donor and recipient age (Figure 3). These findings were consistent with the univariable linear regression results (Supplementary Table S1).

### UEGF Is Associated With Allograft Loss in Multivariable Analysis

**FIGURE 4:**
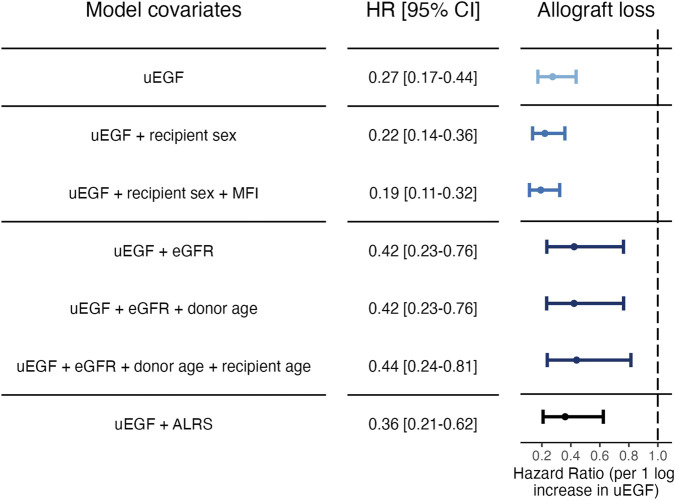
Cause-specific hazard ratios for allograft loss associated with uEGF levels at 3 months post-transplant. Models 1: uEGF alone. Models 2 and 3: step-forward variable selection. Variables were added sequentially based on significance starting from the null model. Model 2: uEGF and recipient sex. Model 3: uEGF, recipient sex and DSA immunodominant MFI at 3 months post-transplant. Models 4 to 6: variable selection based on random forest importance ranking. Variables identified as most associated with uEGF in the random forest analysis were included. Model 4: uEGF and eGFR. Model 5: uEGF, eGFR and donor age. Model 6: uEGF, eGFR, donor age and recipient age. Model 7: uEGF and ALRS (see Supplementary Methods). uEGF, urinary Epidermal Growth Factor; ALRS, Allograft Loss Risk Score as described in Loupy et al. [5]. eGFR, estimated Glomerular Filtration Rate. MFI, anti-HLA donor-specific antibody immunodominant mean fluorescence intensity.

To further investigate the association between uEGF at 3 months and allograft loss, several cause-specific Cox models were constructed using different adjustment strategies: (1) stepwise forward selection, (2) adjustment for variables most associated with uEGF in a random forest analysis, and (3) combination of uEGF and ALRS model. Stepwise forward selection approach identified uEGF as the first covariate added to the model, as it showed the strongest univariable association with the outcome (Supplementary Table S2). Once adjusted for uEGF, eGFR was not significantly associated with allograft loss. The final model included uEGF (adjusted hazard ratio (HR) [95% CI] 0.19 [0.11−0.32]), sex and donor-specific antibodies (DSA) immunodominant mean fluorescence intensity (MFI). When adjusting on the 3 variables most strongly predicting uEGF levels by random forest, or on the ALRS model, uEGF remained significantly associated with the risk of allograft loss (Figure 4 and Supplementary Table S3).

### UEGF Improves Allograft Loss Risk Prediction

**FIGURE 5:**
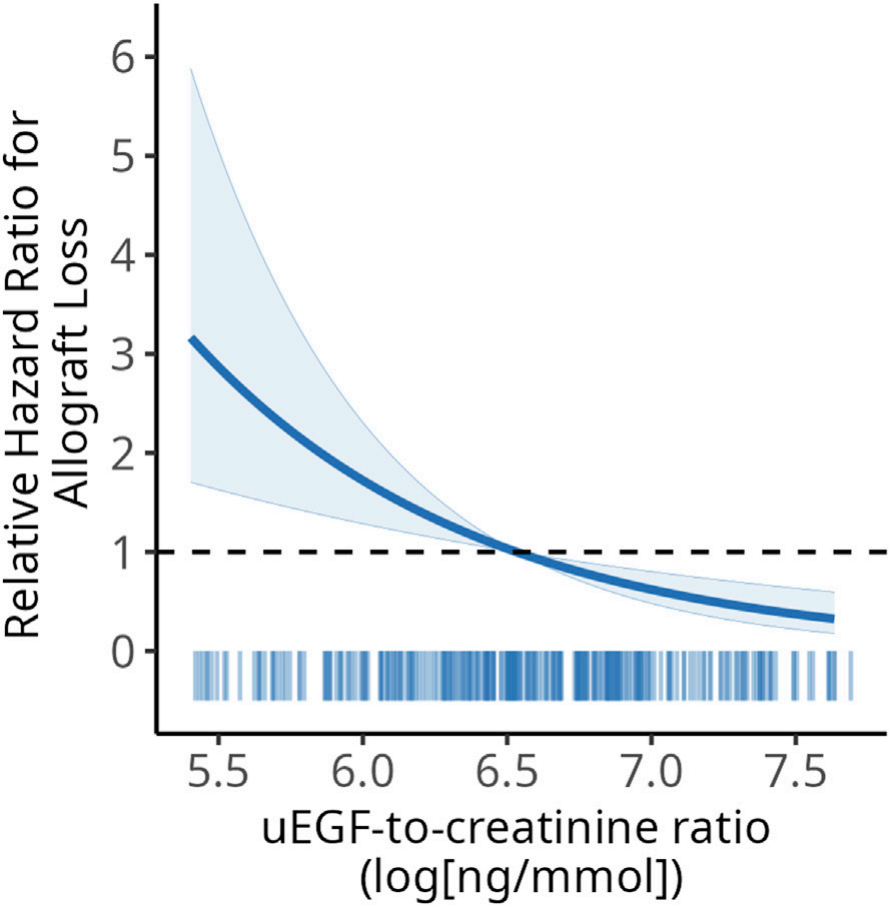
Adjusted hazard ratios of allograft loss associated with uEGF levels. Adjusted on average ALRS value. The shaded area corresponds to 95% confidence interval. uEGF, urinary Epidermal Growth Factor.

Given that uEGF was independently associated with allograft loss, we assessed whether adding it to the ALRS model improved predictive performance. The 7-year timepoint was chosen as it is the longest follow-up duration for which the ALRS score could be computed. The addition of uEGF to the ALRS model improved discrimination (7-year AUC: 0.72 [0.61–0.82] vs. 0.63 [0.53–0.74], p-val = 0.002) and reduced the AIC (394 vs. 383), indicating a better trade-off between model complexity and goodness of fit (Table 2). Similarly, removing uEGF from the stepwise selection model decreased the 7-year AUC (80.35 [76.06–84.64] vs. 65.02 [59.12–70.92], p = 0.004) and increased the AIC (406 vs. 443). In the random forest–based model, removing uEGF did not significantly decrease the 7-year AUC (76.18 [71.63–80.73] vs. 74.73 [69.85–79.61], p = 0.54) but increased the AIC (434 vs. 438) (Supplementary Table S4). The association between uEGF and allograft loss, adjusted on the ALRS score, is visually depicted in Figure 5.

**TABLE 2:** Discrimination performance and model fit of the ALRS model with and without uEGF.

Model	7-year AUC [95% CI]	P-value	AIC
ALRS	0.63 [0.53–0.74]	-	394
ALRS + uEGF	0.72 [0.61–0.82]	0.002	383

uEGF, urinary Epidermal Growth Factor; AIC, Aikake Information Criteria; ALRS, Allograft Loss Risk Score, as described in Loupy et al. [5]. 7-year AUCs were compared as in Blanche et al. [14].

### Internal Validation

**FIGURE 6:**
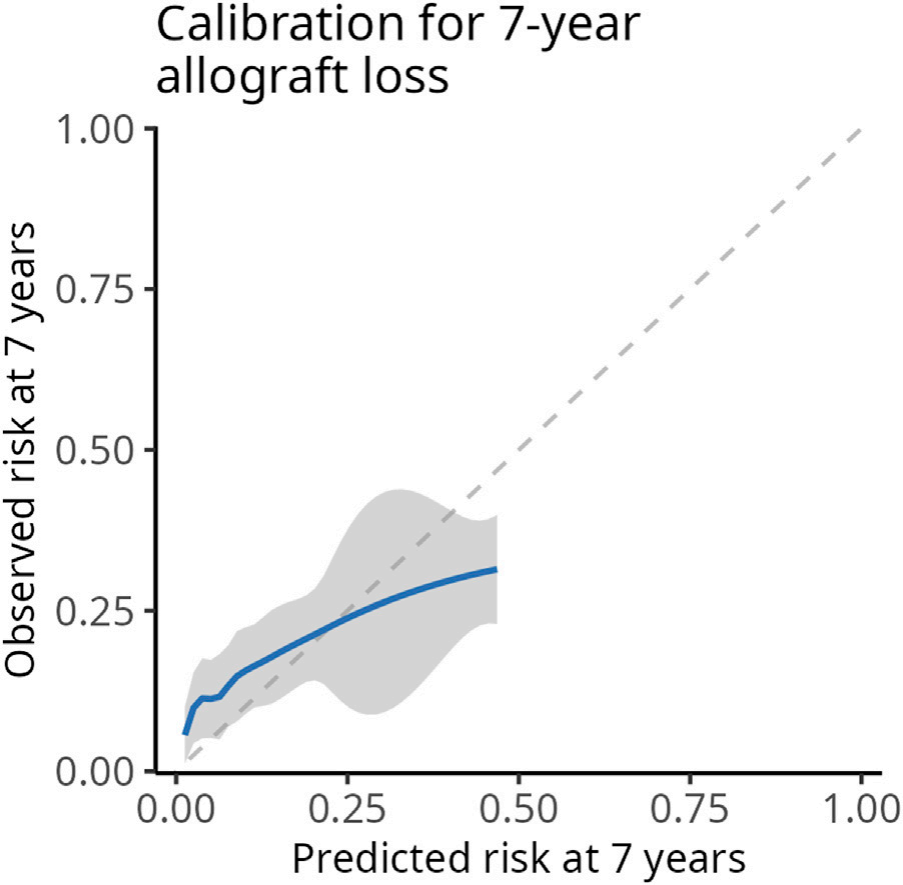
Optimism-corrected calibration plot at 7 years of the ALRS + uEGF model. Average predicted (x-axis) and observed (y-axis) 7- year risks across quantiles of predicted risk. To reflect the original ALRS publication [5], observed 7-year risks were estimated using the Kaplan -Meier method rather than the Aalen -Johansen estimator.

1000 random samples from the original cohort were generated using a bootstrapping procedure. The optimism- corrected 7-year AUC of the uEGF+ALRS model was 0.71 (95% CI 0.68−0.74). The optimism-corrected calibration plot suggested that the model tended to overestimate risk in individuals at higher predicted risk and underestimate it in those at lower predicted risk (Figure 6).

### Temporal Validation of the Association Between uEGF and Allograft Loss

Temporal validation was performed in a retrospective cohort of 203 patients recruited from our center from December 27^th^ 2012 to November 4^th^ 2020 (Supplementary Table S5), to examine whether the association between early post-transplant uEGF levels and long-term allograft loss could be replicated in a temporally distinct population. After a median follow-up of 5.5 years, 30 patients died and 18 experienced allograft loss. UEGF was significantly associated with allograft loss in univariable Cox analysis (HR 0.35 [0.15–0.80], p-value = 0.01), but not once adjusted for eGFR (HR 0.71 [0.26–1.94], p-value = 0.5). The low number of events did not allow for reliable multivariable analysis and performance assessment.

## Discussion

Headway has been made in the past several years to combine predictors of allograft loss like eGFR, proteinuria, allograft scarring or inflammation and anti-HLA DSA profiling into prognostic scores. The current study evaluated the hypothesis that early uEGF, a biomarker of kidney fibrosis, may serve as an independent predictor of long-term allograft loss. Indeed, uEGF measured at 3 months post-transplant was prospectively associated with allograft survival. As anticipated, uEGF levels correlated with markers of chronic kidney injury such as eGFR, donor age, and interstitial fibrosis. Nevertheless, uEGF remained independently associated with allograft loss after adjusting for these factors or for the current reference prediction model. Moreover, adding uEGF to this model improved its predictive performance. While results were robust to internal validation, temporal validation did not show an independent association of uEGF with allograft loss.

UEGF was measured at 3 months post-transplant, together with a screening biopsy. The rationale for early identification of patients at high risk of reduced long-term allograft survival is to target them with dedicated therapeutic strategies apt to modify their eGFR trajectory. Although several kidney donor characteristics are informative regarding transplantation outcomes, risk evaluation in the very first weeks post-surgery may be confounded by acute events: so far, urinary biomarkers measured at the time of donation provided limited insight in allograft function prediction [17]. Similarly, in the ALRS derivation cohort, day 0 parameters were not associated with allograft survival after adjustment for post-transplant parameters, which were mostly evaluated within the first 18 months post- transplant.

As uEGF is strongly associated with markers of chronic kidney damage, we may wonder the extent to which uEGF is a marker of functional nephron mass or provides independent information *per se*. Yepes-Calderon et al. [10] suggested a link between uEGF and early allograft loss, but the interpretation of their results was limited by important heterogeneity in risk evaluation timing, the lack of histological data to properly adjust allograft loss prediction and shorter follow-up interval. We were able to thoroughly evaluate the relationship between uEGF and the other markers of chronic kidney damage including histological ones, and assess their association with long-term allograft loss. At 3 months post-transplant, none of them were independently associated with allograft loss when adjusted on uEGF. Taken together, these results suggest molecular mechanisms related to kidney fibrosis may be detectable early and carry prognostic value for long-term allograft function. Lower uEGF may reflect ongoing fibrotic processes triggered by peri-transplant injury or an underlying susceptibility to future fibrosis progression. However, the lack of temporal replication—evidenced by the absence of an independent association between uEGF and allograft loss in the validation cohort—underscores the need to further investigate the consistency and robustness of uEGF as a prognostic biomarker.

This study has several strengths. We were able to assess uEGF prognostic value in a well-phenotyped, homogenous cohort of transplant recipients, with a median follow-up time of nearly 9 years. Extensive availability of allograft histology at the time of uEGF measurement allowed us to better understand the interrelations between uEGF and the other markers of chronic kidney damage, as well as to include them in our multivariable models. The association between uEGF and graft failure was internally validated and robust to adjustment for the ALRS model.

This study has several limitations. First, uEGF measurements were not repeated, and data on how uEGF levels fluctuate over time are lacking. Although the addition of uEGF improved the predictive performance of the ALRS model in our cohort, it is important to note that the baseline performance of the ALRS was substantially lower than that reported in its original derivation and validation studies. Several factors may account for this discrepancy. Notably, our cohort consisted exclusively of patients assessed at 3 months post-transplant, an earlier time point than the one used in the development of the ALRS score. At 3 months, important prognostic events and risk factors may not yet have fully manifested, potentially limiting the model’s ability to stratify long-term risk. Furthermore, the relatively small cohort size limited the statistical power and increased the risk of overfitting. It restricted the number of covariates that could be reliably included, potentially overlooking important confounders. Additionally, it may have contributed to less precise effect estimates and limited the generalizability of our findings to broader transplant populations.

Altogether, our findings contribute to the ongoing discussion of whether uEGF offers prognostic information beyond established markers such as eGFR, or integrated prognostic models like the ALRS. While uEGF shows promise as an independent predictor, further studies in larger, diverse cohorts are needed to clarify its added value and potential role in clinical risk stratification.

## Data Availability

The datasets analyzed during the current study are available from the corresponding author on reasonable request. Requests will be reviewed to ensure compliance with ethical guidelines and data protection regulations, and a data-sharing agreement may be required.
