# Corrigendum: Early Post-Transplant Urinary EGF as a Potential Predictor of Long-Term Allograft Loss in Kidney Transplant Recipients

**DOI:** 10.3389/ti.2026.16117

**Published:** 2026-01-30

**Authors:** Antoine Créon, Lise Morin, Virginia Garcia, Laila Aouni, Marion Rabant, Fabiola Terzi, Dany Anglicheau

**Affiliations:** 1 Department of Nephrology and Kidney Transplantation, Necker Hospital, AP-HP, Paris, France; 2 Department of Medical Epidemiology and Biostatistics, Karolinska Institutet, Stockholm, Sweden; 3 Université Paris Cité, INSERM U1151, CNRS UMR8253, Institut Necker Enfants Malades (INEM), Paris, France; 4 Department of Pathology, Necker Hospital, AP-HP, Paris, France

**Keywords:** fibrosis, kidney transplant failure, allograft dysfunction, survival analysis, epidermal growth factor receptor

In the original article, we referred throughout the manuscript to a prognostic score described in Loupy et al., *BMJ*, 2019 (1) using the name “iBox”. After publication, the authors of the original score informed us that this score is patented and that the proprietary instrument marketed under the name “iBox” may not be identical in every respect to our independently implemented algorithm.

To avoid any confusion, the terminology used in our article has been updated to reflect that we implemented the score as described in its original publication, without access to the patented version.

These wording changes are editorial in nature and concern only the terminology used to describe the prognostic score.

(1) Loupy A, Aubert O, Orandi BJ, et al. Prediction system for risk of allograft loss in patients receiving kidney transplants: international derivation and validation study. *BMJ*. 2019; 366:l4923. doi:10.1136/bmj.l4923

The changes made are detailed below:

Corrections have been made to the **Abstract**.

“Model performance was compared to an existing prediction model using 7-year time-dependent AUC and Akaike Information Criterion (AIC), with internal validation via bootstrap resampling. Temporal validation was performed in an independent cohort of 203 patients. uEGF correlated with markers of chronic injury, including eGFR, donor age, and interstitial fibrosis. After a median 8.8-year follow-up, lower uEGF was independently associated with allograft loss (adjusted HR 0.19; 95% CI, 0.11–0.32). Adding uEGF to the existing prediction model improved discrimination (AUC 0.72 vs. 0.63) and reduced AIC (383 vs. 394).”

A correction has been made to the **Materials and methods,** *Statistical Analysis* section, paragraph four.

“To assess the robustness of the association between uEGF and allograft loss, several adjustment strategies were used. First, a stepwise forward selection procedure was applied: starting from a null model, covariates were sequentially added based on statistical significance, with the most strongly associated covariate added at each step. The selection stopped when a maximum of one covariate per 10 events was reached or when no additional covariate met the significance threshold (p < 0.05). Second, a model was built by selecting covariates most associated with uEGF using random forest variable importance rankings. Finally, a model was constructed by adding uEGF to the allograft loss risk score (ALRS) described by Loupy et al., which is the reference model for allograft loss prediction **[5]**.”

In the same section corrections were made to paragraph 5.

“The models’ discrimination ability was evaluated using the time-dependent area under the curve (AUC) at 7 years, as risks of allograft loss beyond 7 years could not be derived from the original ALRS publication (see **Supplementary Methods**). Discrimination was assessed for both the ALRS model and the extended model including uEGF, and their 7-year AUC was compared as in Blanche et al. **[15]**. Confidence intervals for the AUC were obtained using the estimated standard error of the AUC and assuming approximate normality. The Akaike Information Criterion (AIC) was also used to compare model fit, with lower AIC values indicating a better balance between complexity and goodness of fit. Harrell’s C-index was not used, as it may be less appropriate in this setting where risk predictions are made at a specific time point **[16]**. To account for overfitting, internal validation was performed using 1,000 bootstrap resamples. Discrimination and calibration were optimism-corrected, with the latter assessed visually using a calibration plot comparing predicted and observed 7-year risks across quantiles of predicted risk. To reflect the original ALRS publication, observed 7-year risks were estimated using the Kaplan–Meier method rather than the Aalen–Johansen estimator when assessing calibration. Only complete cases were used in the analysis. Data management, statistical analyses and graphics were performed using R software 4.1.2.”

Corrections have been made to the **Results**, *UEGF Is Associated With Allograft Loss in Multivariable Analysis* section.

“To further investigate the association between uEGF at 3 months and allograft loss, several cause-specific Cox models were constructed using different adjustment strategies: (1) stepwise forward selection, (2) adjustment for variables most associated with uEGF in a random forest analysis, and (3) combination of uEGF and ALRS model. Stepwise forward selection approach identified uEGF as the first covariate added to the model, as it showed the strongest univariable association with the outcome (**Supplementary Table S2**). Once adjusted for uEGF, eGFR was not significantly associated with allograft loss. The final model included uEGF (adjusted hazard ratio (HR) [95% CI] 0.19 [0.11–0.32]), sex and donor-specific antibodies (DSA) immunodominant mean fluorescence intensity (MFI). When adjusting on the 3 variables most strongly predicting uEGF levels by random forest, or on the ALRS model, uEGF remained significantly associated with the risk of allograft loss (Figure 4 and **Supplementary Table S3**).”

Corrections have been made to the **Results**, *UEGF Improves Allograft Loss Risk Prediction* section. The sentence “The 7-year timepoint was chosen as it is the longest follow-up duration for which the ALRS score could be computed.” was also omitted in the originally published article.

“Given that uEGF was independently associated with allograft loss, we assessed whether adding it to the ALRS model improved predictive performance. The 7-year timepoint was chosen as it is the longest follow-up duration for which the ALRS score could be computed. The addition of uEGF to the ALRS model improved discrimination (7-year AUC: 0.72 [0.61–0.82] vs. 0.63 [0.53–0.74], p-val = 0.002) and reduced the AIC (394 vs. 383), indicating a better trade-off between model complexity and goodness of fit (Table 2). Similarly, removing uEGF from the stepwise selection model decreased the 7-year AUC (80.35 [76.06–84.64] vs. 65.02 [59.12–70.92], p = 0.004) and increased the AIC (406 vs. 443). In the random forest–based model, removing uEGF did not significantly decrease the 7-year AUC (76.18 [71.63–80.73] vs. 74.73 [69.85–79.61], p = 0.54) but increased the AIC (434 vs. 438) (**Supplementary Table S4**). The association between uEGF and allograft loss, adjusted on the ALRS score, is visually depicted in **Figure 5**.”

A correction has been made to the **Results,** *Internal Validation* section.

“1000 random samples from the original cohort were generated using a bootstrapping procedure. The optimism-corrected 7-year AUC of the uEGF+ALRS model was 0.71 (95% CI 0.68–0.74). The optimism-corrected calibration plot suggested that the model tended to overestimate risk in individuals at higher predicted risk and underestimate it in those at lower predicted risk (**Figure 6**).”

A correction has been made to the **Discussion,** paragraph 2.

“UEGF was measured at 3 months post-transplant, together with a screening biopsy. The rationale for early identification of patients at high risk of reduced long-term allograft survival is to target them with dedicated therapeutic strategies apt to modify their eGFR trajectory. Although several kidney donor characteristics are informative regarding transplantation outcomes, risk evaluation in the very first weeks post-surgery may be confounded by acute events: so far, urinary biomarkers measured at the time of donation provided limited insight in allograft function prediction **[17]**. Similarly, in the ALRS derivation cohort, day 0 parameters were not associated with allograft survival after adjustment for post-transplant parameters, which were mostly evaluated within the first 18 months post-transplant.”

A correction has been made to the **Discussion**, paragraph 4.

“This study has several strengths. We were able to assess uEGF prognostic value in a well-phenotyped, homogenous cohort of transplant recipients, with a median follow-up time of nearly 9 years. Extensive availability of allograft histology at the time of uEGF measurement allowed us to better understand the interrelations between uEGF and the other markers of chronic kidney damage, as well as to include them in our multivariable models. The association between uEGF and graft failure was internally validated and robust to adjustment for the ALRS model.”

Corrections have been made to the **Discussion**, paragraph 5.

“This study has several limitations. First, uEGF measurements were not repeated, and data on how uEGF levels fluctuate over time are lacking. Although the addition of uEGF improved the predictive performance of the ALRS model in our cohort, it is important to note that the baseline performance of the ALRS was substantially lower than that reported in its original derivation and validation studies. Several factors may account for this discrepancy. Notably, our cohort consisted exclusively of patients assessed at 3 months post-transplant, an earlier time point than the one used in the development of the ALRS score. At 3 months, important prognostic events and risk factors may not yet have fully manifested, potentially limiting the model’s ability to stratify long-term risk. Furthermore, the relatively small cohort size limited the statistical power and increased the risk of overfitting. It restricted the number of covariates that could be reliably included, potentially overlooking important confounders. Additionally, it may have contributed to less precise effect estimates and limited the generalizability of our findings to broader transplant populations.”

A correction has been made to the **Discussion,** paragraph 6.

“Altogether, our findings contribute to the ongoing discussion of whether uEGF offers prognostic information beyond established markers such as eGFR, or integrated prognostic models like the ALRS. While uEGF shows promise as an independent predictor, further studies in larger, diverse cohorts are needed to clarify its added value and potential role in clinical risk stratification.”

A correction has been made to Table 2. The corrected Table 2 appears below.

**TABLE 2:** Discrimination performance and model fit of the ALRS model with and without uEGF.

Model	7-year AUC [95% CI]	P-value	AIC
ALRS	0.63 [0.53–0.74]	-	394
ALRS + uEGF	0.72 [0.61–0.82]	0.002	383

uEGF, urinary Epidermal Growth Factor; AIC, Aikake Information Criteria; ALRS, Allograft Loss Risk Score, as described in Loupy et al. **[5]**. 7-year AUCs were compared as in Blanche et al. **[14]**.

A correction was made to Figure 4 and its caption. The corrected figure four and caption appear below.

**FIGURE 4:**
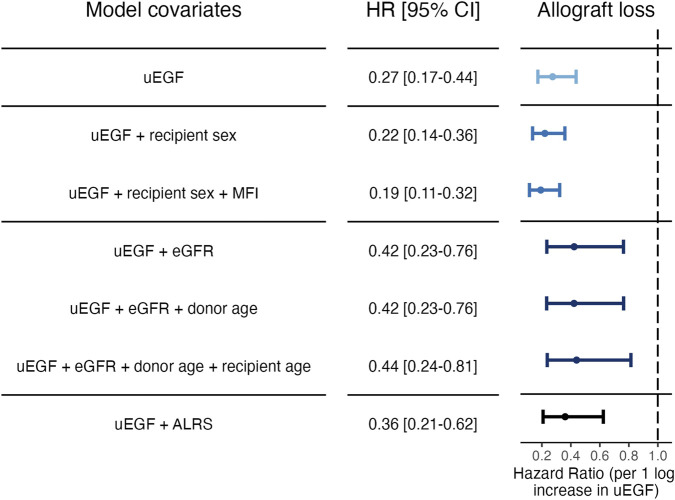
Cause‐specific hazard ratios for allograft loss associated with uEGF levels at 3 months post‐transplant. Models 1: uEGF alone. Models 2 and 3: step‐forward variable selection. Variables were added sequentially based on significance starting from the null model. Model 2: uEGF and recipient sex. Model 3: uEGF, recipient sex and DSA immunodominant MFI at 3 months post‐transplant. Models 4 to 6: variable selection based on random forest importance ranking. Variables identified as most associated with uEGF in the random forest analysis were included. Model 4: uEGF and eGFR. Model 5: uEGF, eGFR and donor age. Model 6: uEGF, eGFR, donor age and recipient age. Model 7: uEGF and ALRS (see **Supplementary Methods**). uEGF, urinary Epidermal Growth Factor; ALRS, Allograft Loss Risk Score as described in Loupy et al. **[5]**. eGFR, estimated Glomerular Filtration Rate. MFI, anti‐HLA donor‐specific antibody immunodominant mean fluorescence intensity.

Cause-specific hazard ratios for allograft loss associated with uEGF levels at 3 months post-transplant. Models 1: uEGF alone. Models 2 and 3: step‐forward variable selection. Variables were added sequentially based on significance starting from the null model. Model 2: uEGF and recipient sex. Model 3: uEGF, recipient sex and DSA immunodominant MFI at 3 months post‐transplant. Models 4 to 6: variable selection based on random forest importance ranking. Variables identified as most associated with uEGF in the random forest analysis were included. Model 4: uEGF and eGFR. Model 5: uEGF, eGFR and donor age. Model 6: uEGF, eGFR, donor age and recipient age. Model 7: uEGF and ALRS (see **Supplementary Methods**). uEGF, urinary Epidermal Growth Factor; ALRS, Allograft Loss Risk Score as described in Loupy et al. **[5]**. eGFR, estimated Glomerular Filtration Rate. MFI, anti-HLA donor‐specific antibody immunodominant mean ‘fluorescence intensity’.

A correction was made to the Legend of **Figure 5** the correct legend appears below.

“Adjusted hazard ratios of allograft loss associated with uEGF levels. Adjusted on average ALRS value. The shaded area corresponds to 95% confidence interval. uEGF, urinary Epidermal Growth Factor.”

A correction was made to the Legend of **Figure 6** the correct legend appears below.

“Optimism-corrected calibration plot at 7 years of the ALRS + uEGF model. Average predicted (x-axis) and observed (y-axis) 7-year risks across quantiles of predicted risk. To reflect the original ALRS publication **[5]**, observed 7-year risks were estimated using the Kaplan–Meier method rather than the Aalen–Johansen estimator.”

Corrections have been made to the **Supplementary Material**.

A correction has been made to the **Supplementary Methods**, section one heading.

“Allograft Loss Risk Score (ALRS) model”

A correction has been made to the **Supplementary Methods**, *7-year allograft survival probability* section.

“The allograft survival probability S(t) is computed from an expression of the form:

$S\left(t\right)={S_{0}\left(t\right)}^{exp\left(LP\right)}$

With $S_{0}\left(t\right)$ the baseline survival at time t in the ALRS derivation cohort, which is not available in the original publication. However, it can be calculated at 7 years from the example provided in **Supplementary Figure B** of the original manuscript (1), as all parameters of the linear predictor and the predicted 7-year survival are given.”

A correction has been made to the **Supplementary Methods**, *Section 3*, and its heading.

“7-year allograft survival probability of the ALRS + uEGF model

The 7-year survival probability of the combined ALRS+uEGF model was calculated from the above baseline survival, and the sum of the beta coefficients of uEGF and the ALRS model.”

Corrections have been made to **Supplementary Table 3**:

**Supplementary Table 3:** Number of individuals at risk and number of outcomes in the Cox models.

**Table:** 

Model	Number at risk	Number of events
uEGF	289	43
uEGF + recipient sex	289	43
uEGF + recipient sex + MFI	276	42
uEGF + eGFR	289	43
uEGF + eGFR + donor age	289	43
uEGF + eGFR + donor age + recipient age	289	43
uEGF + ALRS	257	38

uEGF, urinary Epidermal Growth Factor; eGFR, estimated Glomerular Filtration Rate; MFI, anti-HLA donor-specific antibody immunodominant mean fluorescence intensity; ALRS, Allograft Loss Risk Score.

The authors apologize for this error and state that this does not change the scientific conclusions of the article in any way. The original article has been updated.

