# Supplementary material for: Early Post-Transplant Urinary EGF as a Potential Predictor of Long-Term Allograft Loss in Kidney Transplant Recipients
Source: Transpl Int. 2025 Oct 20;38:15061. doi: 10.3389/ti.2025.15061 (PMC12580079; doi:10.3389/ti.2025.15061)
Supplement: Supplementary file 1 [file DataSheet1.docx]

**Early post-transplant urinary EGF as a potential predictor of long-term allograft loss in kidney transplant recipients – supplementary material**

Antoine Créon^1,2^, Lise Morin^1^, Virginia Garcia^3^, Laila Aouni^1^, Marion Rabant^3,4^, Fabiola Terzi^3^, Dany Anglicheau^1,3^

^1^Department of Nephrology and Kidney Transplantation, Necker Hospital, AP-HP, Paris, France

^2^Department of Medical Epidemiology and Biostatistics, Karolinska Institutet, Stockholm, Sweden

^3^Université Paris Cité, INSERM U1151, CNRS UMR8253, Institut Necker Enfants Malades (INEM), Paris, France.

^4^Department of Pathology, Necker Hospital, AP-HP, Paris, France

**Corresponding author**: Antoine Créon ([antoine.creon@ki.se](mailto:antoine.creon@ki.se))

Contents

[Capsule sentence summary 2](#_Toc219308295)

[Supplementary methods 3](#_Toc219308296)

[ALRS model 3](#_Toc219308297)

[Supplementary figures 4](#_Toc219308298)

[Supplementary figure 1**:** uEGF distribution in the cohort after log-transformation 4](#_Toc219308299)

[Supplementary tables 5](#_Toc219308300)

[Supplementary table 1: Univariable linear regression analysis of covariates predicting urinary EGF levels at 3 months post-transplant. 6](#_Toc219308301)

[Supplementary table 2: univariable cause-specific Cox regression analysis for allograft failure 9](#_Toc219308302)

[Supplementary table 3: number of individuals at risk and number of outcomes in the Cox models 12](#_Toc219308303)

[Supplementary table 4: discrimination performance and model fit of the multivariable models with and without uEGF. 13](#_Toc219308304)

[Supplementary table 5: validation cohort baseline characteristics. 14](#_Toc219308305)

[References 15](#_Toc219308306)

# Capsule sentence summary

Urinary EGF may refine long-term risk prediction after kidney transplantation and improve existing models, but inconclusive temporal validation underscores the need for confirmation in larger studies.

# Supplementary methods

## ALRS model

**Linear predictor (LP)**

$$LP=log\left( 1.08 \right)*t+$$

$$log\left( 0.96 \right)*eGFR+$$

$$log\left( 1.51 \right)*log\left( uprot \right)+$$

$$log\left( 1.14 \right)*IFTA_{2}+log\left( 1.39 \right)*IFTA_{3}+$$

$$log\left( 1.45 \right)*MVI_{3-4}+log\left( 1.83 \right)*MVI_{5-6}+$$

$$log\left( 1.34 \right)*IIT_{\geq3}+$$

$$log\left( 1.47 \right)*cg_{\geq1}+$$

$$log\left( 1.25 \right)*MFI_{500-3000}+log\left( 1.72 \right)*MFI_{3000-6000}+log\left( 2.05 \right)*MFI_{\geq6000}$$

With:

- t: time from transplant to evaluation (years)
- eGFR: glomerular filtration rate (ml/min/1.73m^2^)
- uprot: urine protein/creatinine ratio (g/g)
- IFTA: interstitial fibrosis/tubular atrophy, categorized in 0-1/2/3
- MVI: microvascular inflammation (g+ptc), categorized in 0-2/3-4/5-6
- IIT: interstitial inflammation and tubulitis (i+t), categorized in 0-2/$\geq3$
- cg: transplant glomerulopathy, categorized as 0/$\geq1$
- MFI: Anti-HLA donor specific antibody mean fluorescence intensity, categorized as <500/500-3000/3000-6000/$\geq$6000

**7-year allograft survival probability**

The allograft survival probability S(t) is computed from an expression of the form:

$S\left( t \right)=S_{0}\left( t \right)^{\boldsymbol{exp}\left( \boldsymbol{LP} \right)}$

With $S_{0}\left( t \right)$ the baseline survival at time t in the ALRS derivation cohort, which is not available in the original publication. However, it can be calculated at 7 years from the example provided in Supplementary Figure B of the original manuscript (1), as all parameters of the linear predictor and the predicted 7-year survival are given.

**7-year allograft survival probability of the ALRS + uEGF model**

The 7-year survival probability of the combined ALRS+uEGF model was calculated from the above baseline survival, and the sum of the beta coefficients of uEGF and the ALRS model.

# Supplementary figures

## Supplementary figure 1**:** uEGF distribution in the cohort after log-transformation


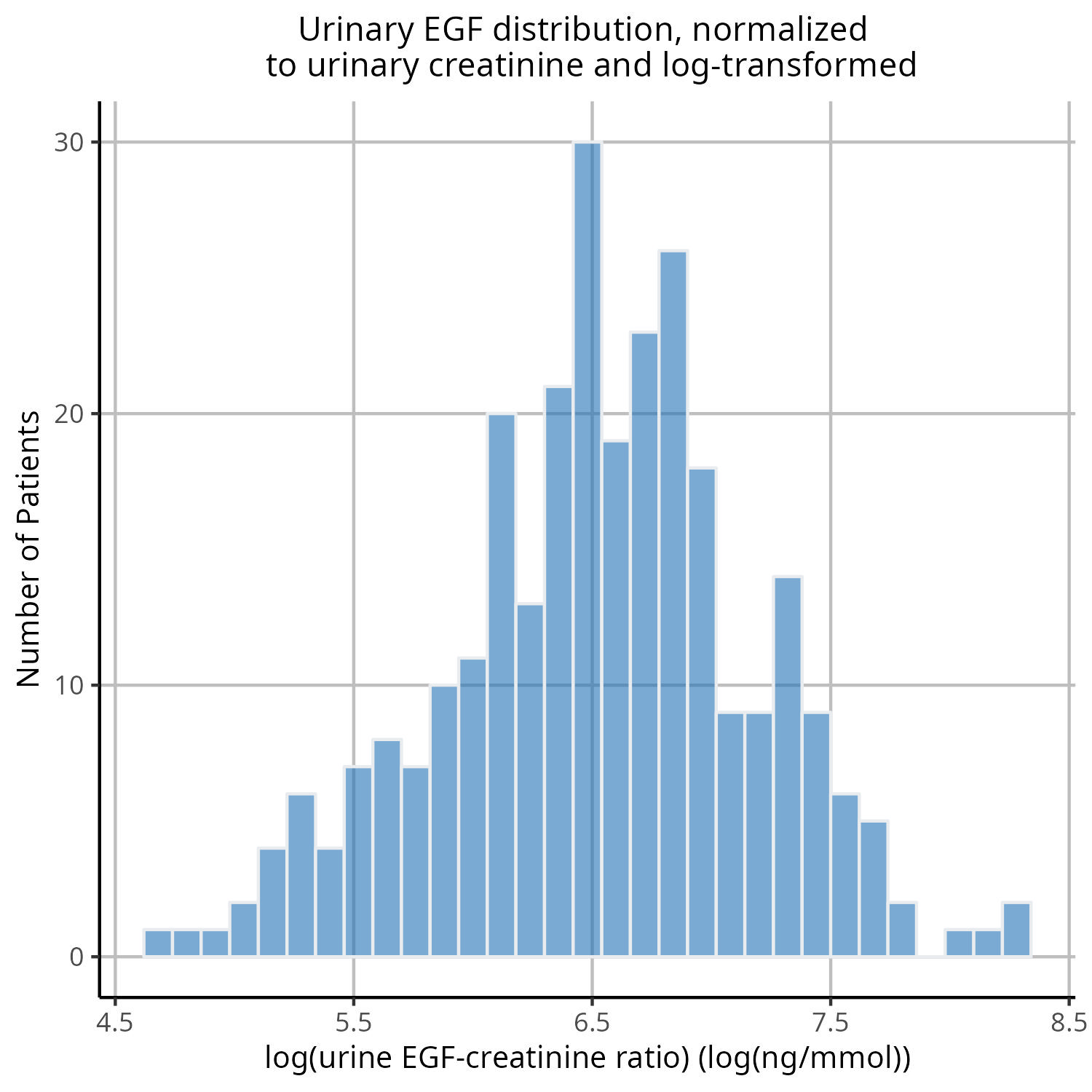


# Supplementary tables

## Supplementary table 1: Univariable linear regression analysis of covariates predicting urinary EGF levels at 3 months post-transplant.

| **Characteristic** | **N** | **Beta** | **95% CI***^1^* | **p-value** |
| --- | --- | --- | --- | --- |
| **Donor age** | 290 | -0.01 | -0.02, -0.01 | <0.001 |
| **Donor type** | 290 |  |  | <0.001 |
| Standard-Criteria |  | — | — |  |
| Expanded-Criteria |  | -0.42 | -0.58, -0.25 |  |
| Living Donor |  | 0.03 | -0.16, 0.22 |  |
| **Recipient age** | 290 | 0.00 | -0.01, 0.00 | 0.77 |
| **Recipient sex** | 290 |  |  | <0.001 |
| Male |  | — | — |  |
| Female |  | 0.33 | 0.18, 0.48 |  |
| **Cause of end stage renal disease** | 290 |  |  | 0.86 |
| Diabetes |  | — | — |  |
| Glomerulonephritis |  | 0.03 | -0.24, 0.31 |  |
| Hypertensive |  | 0.06 | -0.31, 0.44 |  |
| Tubulo-interstitial |  | 0.05 | -0.29, 0.40 |  |
| Autosomal dominant polycystic kidney disease |  | 0.12 | -0.18, 0.42 |  |
| Unknown |  | 0.13 | -0.16, 0.41 |  |
| Other |  | -0.06 | -0.37, 0.26 |  |
| **Recipient body mass index** | 290 | -0.01 | -0.02, 0.01 | 0.30 |
| **Prior kidney transplant** | 290 |  |  | 0.057 |
| No |  | — | — |  |
| Yes |  | -0.21 | -0.42, 0.01 |  |
| **Cold ischaemia time (min)** | 287 | 0.00 | 0.00, 0.00 | 0.008 |
| **HLA A/B/DR mismatch** | 290 | -0.03 | -0.09, 0.02 | 0.24 |
| **ABO compatibility** | 290 |  |  | 0.36 |
| Yes |  | — | — |  |
| No |  | 0.11 | -0.13, 0.36 |  |
| **Pre-existing anti-HLA donor-specific antibody** | 290 |  |  | 0.36 |
| MFI<500 |  | — | — |  |
| MFI 500-1000 |  | 0.06 | -0.12, 0.24 |  |
| MFI 1000-3000 |  | -0.16 | -0.39, 0.06 |  |
| MFI > 3000 |  | 0.06 | -0.26, 0.39 |  |
| **DSA Immunodominant MFI at M3** | 277 |  |  | 0.36 |
| <500 |  | — | — |  |
| 500-3000 |  | -0.11 | -0.27, 0.05 |  |
| 3000-6000 |  | -0.11 | -0.50, 0.27 |  |
| >6000 |  | -0.26 | -0.60, 0.09 |  |
| **Induction immunosuppression** | 290 |  |  | 0.71 |
| Anti-thymocyte globulin |  | — | — |  |
| Basiliximab |  | 0.04 | -0.11, 0.20 |  |
| No induction |  | -0.11 | -0.51, 0.29 |  |
| **Maintenance immunosuppression** | 290 |  |  | 0.72 |
| Steroids, Mycophenolate Mofetil and Ciclosporine |  | — | — |  |
| Steroids, Mycophenolate Mofetil and Tacrolimus |  | 0.07 | -0.12, 0.25 |  |
| Steroids, Mycophenolate Mofetil and Everolimus |  | -0.10 | -0.86, 0.65 |  |
| **Delayed graft function** | 290 |  |  | <0.001 |
| No |  | — | — |  |
| Yes |  | -0.39 | -0.57, -0.21 |  |
| **Estimated glomerular filtration rate at 3 months (ml/min/1.73m²)** | 290 | 0.02 | 0.02, 0.02 | <0.001 |
| **Proteinuria at 3 months (g/mmol)** | 289 | -0.05 | -0.12, 0.02 | 0.19 |
| **Glomerulitis (g)** | 274 |  |  | 0.74 |
| 0 |  | — | — |  |
| 1 |  | -0.07 | -0.28, 0.14 |  |
| 2 |  | -0.08 | -0.48, 0.31 |  |
| 3 |  | -0.30 | -0.94, 0.35 |  |
| **Interstitial inflammation (i)** | 274 |  |  | 0.53 |
| 0 |  | — | — |  |
| 1 |  | 0.20 | -0.21, 0.61 |  |
| 2 |  | -0.23 | -0.97, 0.51 |  |
| **Total interstitial inflammation (ti)** | 274 |  |  | 0.045 |
| 0 |  | — | — |  |
| 1 |  | -0.43 | -0.78, -0.08 |  |
| 2 |  | -0.25 | -0.77, 0.27 |  |
| 3 |  | -0.57 | -1.5, 0.33 |  |
| **Tubulitis (t)** | 274 |  |  | 0.66 |
| 0 |  | — | — |  |
| 1 |  | -0.20 | -0.55, 0.15 |  |
| 2 |  | 0.02 | -0.47, 0.51 |  |
| 3 |  | 0.09 | -0.24, 0.42 |  |
| **Intimal arteritis (v)** | 274 |  |  | 0.10 |
| 0 |  | — | — |  |
| 1 |  | 0.33 | -0.57, 1.2 |  |
| 3 |  | 1.3 | 0.05, 2.6 |  |
| **Peritubular capillaritis (ptc)** | 274 |  |  | 0.83 |
| 0 |  | — | — |  |
| 1 |  | -0.03 | -0.31, 0.24 |  |
| 2 |  | 0.08 | -0.30, 0.46 |  |
| 3 |  | -0.30 | -1.0, 0.45 |  |
| **Interstitial fibrosis (ci)** | 274 |  |  | <0.001 |
| 0 |  | — | — |  |
| 1 |  | -0.40 | -0.57, -0.22 |  |
| 2 |  | -0.68 | -0.91, -0.45 |  |
| 3 |  | -0.59 | -0.83, -0.34 |  |
| **Tubular atrophy (ct)** | 274 |  |  | <0.001 |
| 0 |  | — | — |  |
| 1 |  | -0.36 | -0.54, -0.19 |  |
| 2 |  | -0.66 | -0.89, -0.44 |  |
| 3 |  | -0.55 | -0.81, -0.28 |  |
| **C4d graft deposition (c4d)** | 274 |  |  | 0.079 |
| 0 |  | — | — |  |
| 1 |  | -0.31 | -0.57, -0.05 |  |
| 2 |  | -0.15 | -0.49, 0.18 |  |
| 3 |  | -0.26 | -0.78, 0.27 |  |
| **Vascular Fibrous Intimal Thickening (cv)** | 274 |  |  | <0.001 |
| 0 |  | — | — |  |
| 1 |  | -0.09 | -0.29, 0.11 |  |
| 2 |  | -0.35 | -0.54, -0.17 |  |
| 3 |  | -0.40 | -0.66, -0.14 |  |
| **Glomerular basement membrane double contours (cg)** | 274 |  |  | 0.85 |
| 0 |  | — | — |  |
| 1 |  | 0.05 | -0.52, 0.63 |  |
| **Arteriolar hyalinosis (ah)** | 274 |  |  | <0.001 |
| 0 |  | — | — |  |
| 1 |  | -0.19 | -0.36, -0.03 |  |
| 2 |  | -0.52 | -0.76, -0.28 |  |
| 3 |  | -0.65 | -0.98, -0.31 |  |
| **Polyomavirus associated nephropathy** | 257 |  |  | 0.57 |
| N |  | — | — |  |
| O |  | 0.19 | -0.46, 0.84 |  |
| **Nephropathy recurrence** | 23 |  |  | 0.69 |
| N |  | — | — |  |
| O |  | -0.21 | -1.2, 0.80 |  |
| **Minimum resistance index** | 285 | 0.61 | -0.13, 1.3 | 0.11 |
|  | | | | |

## Supplementary table 2: univariable cause-specific Cox regression analysis for allograft failure

The following variables, without any outcome in some categories, are not shown: i, cg, v, ti, ptc.^1^HR = Hazard Ratio, CI = Confidence Interval

| **Characteristic** | **N** | **HR***^1^* | **95% CI***^1^* | **p-value** |
| --- | --- | --- | --- | --- |
| **Donor age** | 290 | 1.02 | 1.00, 1.04 | 0.014 |
| **Donor type** | 290 |  |  | 0.004 |
| Standard-Criteria |  | — | — |  |
| Expanded-Criteria |  | 2.38 | 1.23, 4.61 |  |
| Living Donor |  | 0.76 | 0.30, 1.90 |  |
| **Recipient age** | 290 | 1.00 | 0.98, 1.02 | 0.92 |
| **Recipient sex** | 290 |  |  | 0.26 |
| Male |  | — | — |  |
| Female |  | 1.39 | 0.78, 2.47 |  |
| **Recipient ethnicity** | 290 |  |  | 0.007 |
| Caucasian |  | — | — |  |
| Black |  | 1.71 | 0.83, 3.52 |  |
| North-african |  | 2.82 | 1.44, 5.51 |  |
| Other |  | 0.00 | 0.00, Inf |  |
| **Cause of end stage renal disease** | 290 |  |  | 0.41 |
| Diabetes |  | — | — |  |
| Glomerulonephritis |  | 0.81 | 0.33, 2.00 |  |
| Hypertensive |  | 0.19 | 0.02, 1.56 |  |
| Tubulo-interstitial |  | 0.61 | 0.16, 2.36 |  |
| Autosomal dominant polycystic kidney disease |  | 0.76 | 0.28, 2.09 |  |
| Unknown |  | 0.40 | 0.14, 1.20 |  |
| Other |  | 0.76 | 0.27, 2.18 |  |
| **Recipient body mass index** | 290 | 0.94 | 0.88, 1.01 | 0.079 |
| **Prior kidney transplant** | 290 |  |  | 0.050 |
| No |  | — | — |  |
| Yes |  | 2.06 | 1.05, 4.06 |  |
| **Cold ischaemia time** | 287 | 1.00 | 1.00, 1.00 | 0.020 |
| **HLA A/B/DR mismatch** | 290 | 0.96 | 0.78, 1.19 | 0.73 |
| **ABO compatibility** | 290 |  |  | 0.11 |
| Yes |  | — | — |  |
| No |  | 0.37 | 0.09, 1.55 |  |
| **Pre-existing anti-HLA donor-specific antibody** | 290 |  |  | >0.99 |
| MFI<500 |  | — | — |  |
| MFI 500-1000 |  | 1.03 | 0.51, 2.08 |  |
| MFI 1000-3000 |  | 1.01 | 0.42, 2.45 |  |
| MFI > 3000 |  | 1.05 | 0.32, 3.45 |  |
| **DSA Immunodominant MFI at M3** | 277 |  |  | 0.17 |
| <500 |  | — | — |  |
| 500-3000 |  | 1.46 | 0.77, 2.79 |  |
| 3000-6000 |  | 1.38 | 0.32, 6.05 |  |
| >6000 |  | 3.54 | 1.28, 9.76 |  |
| **Induction immunosuppression** | 290 |  |  | 0.51 |
| Anti-thymocyte globulin |  | — | — |  |
| Basiliximab |  | 0.77 | 0.43, 1.40 |  |
| No induction |  | 0.44 | 0.06, 3.27 |  |
| **Maintenance immunosuppression** | 290 |  |  | 0.39 |
| Steroids, Mycophenolate Mofetil and Ciclosporine |  | — | — |  |
| Steroids, Mycophenolate Mofetil and Tacrolimus |  | 1.07 | 0.50, 2.29 |  |
| Steroids, Mycophenolate Mofetil and Everolimus |  | 6.17 | 0.76, 50.0 |  |
| **Delayed graft function** | 290 |  |  | 0.041 |
| No |  | — | — |  |
| Yes |  | 1.99 | 1.06, 3.72 |  |
| **Estimated glomerular filtration rate at 3 months (ml/min/1.73m²)** | 290 | 0.96 | 0.94, 0.98 | <0.001 |
| **Proteinuria at 3 months (g/mmol)** | 289 | 1.24 | 0.94, 1.64 | 0.12 |
| **Glomerulitis (g)** | 274 |  |  | 0.64 |
| 0 |  | — | — |  |
| 1 |  | 0.93 | 0.39, 2.21 |  |
| 2 |  | 1.97 | 0.60, 6.44 |  |
| 3 |  | 2.41 | 0.33, 17.7 |  |
| **Tubulitis (t)** | 274 |  |  | 0.96 |
| 0 |  | — | — |  |
| 1 |  | 0.86 | 0.21, 3.56 |  |
| 2 |  | 1.33 | 0.32, 5.55 |  |
| 3 |  | 0.83 | 0.20, 3.43 |  |
| **Interstitial fibrosis (ci)** | 274 |  |  | 0.020 |
| 0 |  | — | — |  |
| 1 |  | 1.94 | 0.91, 4.15 |  |
| 2 |  | 3.37 | 1.50, 7.58 |  |
| 3 |  | 2.60 | 1.02, 6.62 |  |
| **Tubular atrophy (ct)** | 274 |  |  | 0.002 |
| 0 |  | — | — |  |
| 1 |  | 2.20 | 1.03, 4.69 |  |
| 2 |  | 4.73 | 2.21, 10.1 |  |
| 3 |  | 2.41 | 0.80, 7.29 |  |
| **C4d graft deposition (c4d)** | 274 |  |  | 0.51 |
| 0 |  | — | — |  |
| 1 |  | 1.48 | 0.62, 3.56 |  |
| 2 |  | 2.20 | 0.78, 6.24 |  |
| 3 |  | 1.16 | 0.16, 8.52 |  |
| **Vascular Fibrous Intimal Thickening (cv)** | 274 |  |  | 0.069 |
| 0 |  | — | — |  |
| 1 |  | 0.75 | 0.28, 1.97 |  |
| 2 |  | 2.07 | 1.02, 4.19 |  |
| 3 |  | 1.74 | 0.62, 4.89 |  |
| **Arteriolar hyalinosis (ah)** | 274 |  |  | 0.011 |
| 0 |  | — | — |  |
| 1 |  | 1.78 | 0.82, 3.86 |  |
| 2 |  | 3.31 | 1.38, 7.97 |  |
| 3 |  | 5.00 | 1.70, 14.7 |  |
| **IF/TA m3** | 274 |  |  | 0.013 |
| 0/1 |  | — | — |  |
| 2 |  | 2.98 | 1.44, 6.18 |  |
| 3 |  | 2.17 | 0.89, 5.28 |  |
| **i+t m3** | 274 |  |  | 0.77 |
| 0-2 |  | — | — |  |
| >2 |  | 1.17 | 0.42, 3.26 |  |
| **Minimum resistance index** | 285 | 0.63 | 0.03, 15.4 | 0.78 |
| **Urinary EGF-to-creatinine ratio, log transformed (log(ng/mmol))** | 290 | 0.30 | 0.19, 0.47 | <0.001 |
|  | | | | |

## Supplementary table 3: number of individuals at risk and number of outcomes in the Cox models

| Model | Number at risk | Number of events |
| --- | --- | --- |
| uEGF | 289 | 43 |
| uEGF + recipient sex | 289 | 43 |
| uEGF + recipient sex + MFI | 276 | 42 |
| uEGF + eGFR | 289 | 43 |
| uEGF + eGFR + donor age | 289 | 43 |
| uEGF + eGFR + donor age + recipient age | 289 | 43 |
| uEGF + ALRS | 257 | 38 |

ALRS: Allograft Loss Risk Score. uEGF: urinary Epidermal Growth Factor. eGFR: estimated Glomerular Filtration Rate. MFI: anti-HLA donor-specific antibody immunodominant mean fluorescence intensity.

## Supplementary table 4: discrimination performance and model fit of the multivariable models with and without uEGF.

| Model | 7-year AUC [95% CI] | P-value | AIC |
| --- | --- | --- | --- |
| uEGF + recipient sex + MFI | 80.35 [76.06-84.64] | - | 406 |
| recipient sex + MFI | 65.02 [59.12-70.92] | 0.004 | 443 |
| uEGF + eGFR + donor age + recipient age | 76.18 [71.63-80.73] | - | 434 |
| eGFR + donor age + recipient age | 74.73 [69.85-79.61] | 0.54 | 438 |

uEGF: urinary Epidermal Growth Factor. AIC: Aikake Information Criteria. 7-year AUCs were compared as in *Blanche et al.* (2)

## Supplementary table 5: validation cohort baseline characteristics.

| **Baseline Characteristics** | **N = 203***^1^* |
| --- | --- |
| Female | 79 (39%) |
| Recipient age | 49 (14) |
| Donor age | 55 (15) |
| Donor type |  |
| Deceased | 136 (67%) |
| Living | 67 (33%) |
| Estimated glomerular filtration rate at 3 months (ml/min/1.73m²) | 57 (20) |
| Proteinuria at 3 months (g/mmol) | 0.02 (0.01-0.04) |
| Urinary EGF-to-creatinine ratio (ng/mmol) | 1,736 (1,229-2,404) |
| Urinary EGF-to-creatinine ratio, log transformed (log(ng/mmol)) | 7.43 (0.46) |
| Post-transplant follow-up time (years) | 5.50 (3.58-6.65) |
| *^1^*n (%); Mean (SD); Median (Q1-Q3) | |

# References

1. Loupy A, Aubert O, Orandi BJ, Naesens M, Bouatou Y, Raynaud M, et al. Prediction system for risk of allograft loss in patients receiving kidney transplants: international derivation and validation study. BMJ. 2019 Sept 17;366:l4923.

2. Blanche P, Dartigues JF, Jacqmin-Gadda H. Estimating and comparing time-dependent areas under receiver operating characteristic curves for censored event times with competing risks. Stat Med. 2013 Dec 30;32(30):5381–97.
